# Patients with longstanding ulcerative colitis in remission do not have more irritable bowel syndrome-like symptoms than controls

**DOI:** 10.1186/s12876-016-0553-x

**Published:** 2016-11-24

**Authors:** D. Lundgren, J. Rutegård, V. Eklöf, R. Palmqvist, P. Karling

**Affiliations:** 1 Department of Public Health and Clinical Medicine, Division of Medicine, Umeå University, SE-90187 Umeå, Sweden; 2 Department of Surgery and Perioperative Science, Surgery, Umeå University, SE-90187 Umeå, Sweden; 3 Department of Medical Biosciences, Pathology, Umeå University, SE-90187 Umeå, Sweden

**Keywords:** Irritable bowel syndrome, Inflammatory bowel disorder, Ulcerative Colitis

## Abstract

**Background:**

Irritable bowel syndrome (IBS) is more common in patients with ulcerative colitis (UC) than expected. The prevalence of IBS in patients with UC with longstanding disease is not known. We investigated the prevalence of IBS-like symptoms in patients with UC in remission and longstanding disease in comparison to control subjects.

**Methods:**

Sixty-eight patients with UC and 33 patients with hereditary familiar colon cancer and who underwent colonoscopy surveillance were included. Faecal calprotectin (FC), Gastrointestinal Symptoms Rating Scale-Irritable Bowel Syndrome (GSRS-IBS) and Hospital Anxiety and Depression scale were fulfilled prior to endoscopy. UC in remission was define by steroid-free clinical remission, a Mayo Score ≤ 1 on endoscopy, a FC ≤ 200 μg/g and no significant active inflammation on colon biopsies.

**Results:**

Fifty-five UC patients met the criteria for being in remission. The median disease duration was 17 years. The patients with UC in remission tended to have lower scores on total GSRS-IBS score (6 vs 10.5; *p* = 0.062) and lower or equal scores on all specific IBS symptoms in comparison to controls. There was a moderate but significant correlation between diarrhoea scores and FC levels (in the span ≤ 200 μg/g) (rs 0.38; *p* = 0.004) in the UC in remission group.

**Conclusion:**

Patients with UC with longstanding disease and in remission do not have more IBS symptoms than controls. In UC patients in remission the FC level in the lower span showed a moderate correlation to symptoms of diarrhoea.

**Electronic supplementary material:**

The online version of this article (doi:10.1186/s12876-016-0553-x) contains supplementary material, which is available to authorized users.

## Background

Ulcerative colitis (UC) is a chronic inflammatory bowel disease affecting the colon and rectum with an annual incidence of 9–20 per 100 000 in the western population [1, 2]. The natural history of UC is episodic with quiescent periods interspersed with active flare-ups. Although most patients have a decrease in symptoms over time [3] UC is a lifelong condition with potentially negative impacts on the quality of life [4]. Even in patients with UC in objective remission gastrointestinal symptoms are common. A meta-analysis showed that approximately 30% of patients with UC in remission reported irritable bowel syndrome (IBS)-like symptoms [5], which is more than twice as high as the prevalence in a normal population [6]. However there is some evidence that IBS-like symptoms in patients with UC may be due to inflammatory activity that is not apparently seen on endoscopy. For example, UC patients in remission with IBS-like symptoms had higher levels of calprotectin in faeces than patients without IBS symptoms [7]. Most studies investigating the occurrence of IBS-like symptoms in patients with UC in remission have defined remission using either clinical or endoscopic scores [5], and only a few studies have investigated IBS-like symptoms in patients using more strict criteria for remission (based on endoscopic and faecal markers) [8]. A faecal calprotectin (FC) cut-off level of 50–150 μg/g helps to discriminate patients with functional gastrointestinal disease from those patients with inflammatory bowel disorder (IBD) [9, 10].

To date, we lack a consensus on how to define remission in patients with UC using faecal markers. A FC of approximately 120–200 μg/g has been suggested [11–13].

The patients included in previous studies investigating IBS-like symptoms in patients with UC have either had a short duration of the disease or there has been a mixture of patients with short and long duration [5, 8, 14]. Currently, there is a lack of data in the literature regarding what happens over time with IBS symptoms in patients with UC. Do patients with longstanding UC continue to have significant IBS-like symptoms? The primary aim of this study was to compare the prevalence of functional gastrointestinal symptoms including IBS-like symptoms in patients with UC in remission (with a more accurate assessment of remission) and longstanding disease with control subjects. Secondary aims were to investigate whether low-grade inflammation (in the span of a FC < 200 μg/L) correlates with IBS-like symptoms, and to determine whether symptoms of anxiety and depression have an impact on IBS-like symptoms.

## Methods

### Patients and control subjects

Between May 2007 to February 2013 all patients with UC who underwent a surveillance colonoscopy at the endoscopy unit at Umeå University hospital were invited to participate in the study (*n* = 216). At our clinic all patients with an extensive UC are invited to enter a surveillance colonoscopy program for ten years after diagnosis with the aim to detect dysplasia and early cancer. Control subjects were recruited from patients who underwent a surveillance colonoscopy due to hereditary familiar colon cancer.

Both patients and controls were sent an informed consent, questionnaires and a faecal calprotectin test 4–6 weeks before the planned colonoscopy.

### Questionnaires

The Gastrointestinal Symptom Rating Scale-IBS (GSRS-IBS) was used to evaluate IBS-like symptoms. GSRS-IBS is a validated self-assessment instrument for assessing IBS symptoms and it consists of 13 items, each using a Likert scale (0–6 points) spanning from no symptoms to very severe symptoms [15, 16]. The items are grouped into symptom clusters for abdominal pain (two items), bloating (three items), constipation (two items), diarrhoea (four items) and satiety (two items).

In addition to the GSRS-IBS, the study subjects provided information about concurrent medications, previous operations and filled in the Hospital Anxiety and Depression Scale (HADS). HADS is a validated instrument originally designed to screen for symptoms of anxiety and depression among patients with somatic disease [17, 18]. It consists of 7 four-point Likert items each for anxiety and depression. We used the HADS because it exhibits high sensitivity in detecting symptoms of anxiety and depression, it is thoroughly validated, it is easy to fill in, and we have used it in previous research addressing the relationship between anxiety and depression with gastrointestinal symptoms [19].

### Faecal Calprotectin (FC)

Calprotectin is a calcium and zinc binding protein. It is mainly found in the cytoplasm of neutrophils but is also an abundant protein in monocytes and macrophages [20]. FC is resistant to bacterial degradation and is stable in stool at room temperature up to 7 days. FC correlates well with gut inflammation and is an established marker used for screening and for monitoring patients with IBD [21, 22]. In the present study the samples for FC were sent to the accredited Department of Laboratory Medicine, Clinical Chemistry, Umeå University Hospital, and were analysed using the CALPRO® Calprotectin ELISA Test (ALP) according to the manufacturer (Calpro AS, Norway). The study participants were asked to collect the stool sample the day before the bowel preparations for the colonoscopy were scheduled.

### Colonoscopy

The colonoscopy was performed as clinical routine and by different endoscopists. At least 18 biopsies from a minimum of nine locations were taken in each patient, and the specimens were judged by experienced pathologists. All endoscopists and pathologists were blinded by the outcome of questionnaires and the level of the FC.

### Definition of UC remission

The patients were defined as being in remission if all of the following four criteria were met: no present flare-up or steroid treatment, a Mayo Score ≤ 1 on endoscopy [23], a FC ≤ 200 μg/g, and no significant active inflammation on colon biopsies. No significant active inflammation on colon biopsies was defined as inactive (no granulocytic reaction was found either in epithelial or stromal compartments) or mild (existence of pericryptitis and eventually a few granulocytes in crypt, and/or surface epithelium) [24]. Records from the departments of medicine, surgery and endoscopy units were checked to confirm UC diagnosis, to establish clinical remission and to confirm that the colonoscopy was performed in the surveillance program. The records of the control subjects were searched to exclude a coincident gastrointestinal disease (including a change in bowel habits the year before the endoscopy) and that the colonoscopy was performed in the surveillance program.

### Exclusion

**Fig. 1:**
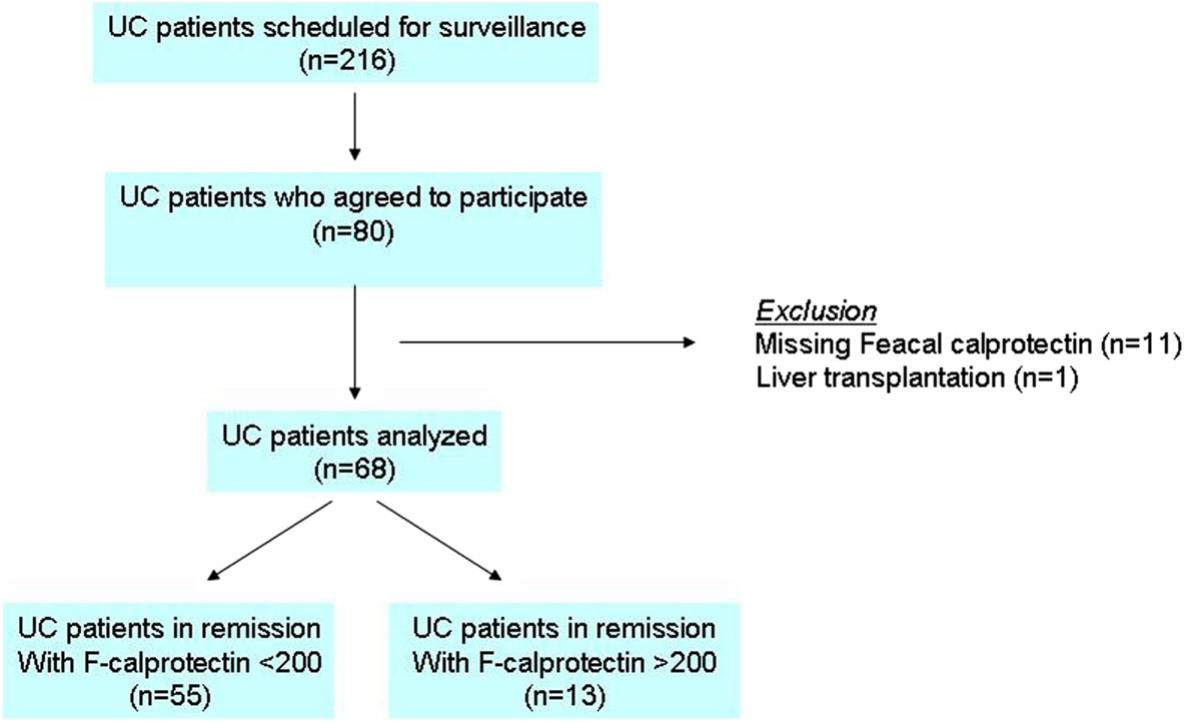
Flow diagram for the selection of the patients with ulcerative colitis

During the study period 216 patients with UC were in the surveillance program, and 80 patients accepted to participate in the study. Eleven patients were excluded due to missing FC tests, one patient was excluded due to a recent liver transplantation, and 13 patients did not meet the criteria for being in remission thus resulting in 55 patients with UC for analysis (Fig. 1). Forty-two control subjects accepted to participate in the study and 9 of these were excluded due to missing FC tests, thus resulting in 33 control subjects for the analysis.

### Statistical analysis

SPSS (version 22) was used for data analysis. Student *t*-test was used for parametric comparisons. Mann-Whitney U non-parametric test was used to evaluate differences between groups. The Spearman non-parametric test was used to evaluate correlations. All tests were 2-sided and with a 5% significance level.

## Results

### Basal characteristics

Table 1 shows the basal characteristics of patients and controls and the Montreal classification [25] for the patients. In comparison to the control subjects, there were significantly fewer women among the patients with UC in remission. The patients with UC in remission had low levels of FC but still significantly higher FC levels than the control subjects.

**Table 1:** Basal characteristics: Patients with ulcerative colitis in remission and controls

	Ulcerative colitis in remission (*n* = 55)	Controls (*n* = 33)	*p*-value
Mean age years (SD)	49 (14.2)	52 (10.2)	0.27
Women	45%	60%	0.03*
Median disease duration in years (IQR)	17.5 (17)		
Montreal classification:			
A1	18%		
A2	67%		
A3	15%		
E1	0%		
E2	20%		
E3	80%		
5-ASA	85%		
Immune modulators	20%		
Biologics	2%		
Median fecal calprotectin μg/g (IQR)	37 (42)	21 (18)	<0.02*

*Statistically significant

Ulcerative colitis in remission was defined by steroid-free clinical remission, a Mayo Score ≤1 on endoscopy, a fecal calprotectin ≤200 μg/g and no significant active inflammation on colon biopsies

### IBS-like symptoms in patients with UC and controls

There was no difference in the total GSRS-IBS scores between patients with UC in remission and controls (Table 2). Symptoms of constipation were significantly higher in the controls in comparison to the UC patients in remission but there were no differences in the other symptom clusters.

**Table 2:** Irritable bowel syndrome-like symptoms and symptoms of anxiety/depression in patients with ulcerative colitis versus control subjects

	Ulcerative colitis in remission (*n* = 55)	Controls (*n* = 33)	*p*-value
Median GSRS-IBS scores			
Total score	6.0	10.5	0.062
Abdominal pain	0.5	0.5	0.57
Bloating	1.0	1.0	0.19
Diarrhoea	0.5	0.5	0.38
Constipation	0.0	0.5	0.048*
Satiety	0.0	0.0	0.14
Median HADS scores			
Anxiety	4.0	4.0	0.52
Depression	2.0	2.0	0.99

*Statistically significant

For the specific IBS symptoms the table presents the total score for each cluster divided by number of items for each cluster

Ulcerative colitis in remission was defined by steroid-free clinical remission, a Mayo Score ≤ 1 on endoscopy, a fecal calprotectin ≤ 200 μg/g and no significant active inflammation on colon biopsies.

*GSRS-IBS* Gastrointestinal symptoms rating scale for irritable bowel syndrome, *HADS* Hospital anxiety and depression scale

### Correlations between faecal calprotectin and IBS-like symptoms

There was no significant correlation between FC levels and total GSRS-IBS score in patients with UC in remission (Table 3). When analysing the different symptom clusters there was a significant correlation between FC and symptoms of diarrhoea in the patients with UC in remission. In the control group there were no statistically significant correlations between FC and total GSRS-IBS scores or any of the symptom clusters in the control group.

**Table 3:** Correlations between irritable bowel syndrome-like symptoms and faecal calprotectin in patients with ulcerative colitis and in control subjects

	Ulcerative colitis in remission (*n* = 55) rs (*p*-value)	Controls (*n* = 33) rs (*p*-value)
Total GSRS-IBS score	0.214 (0.124)	0.190 (0.115)
Abdominal pain	0.003 (0.985)	0.084 (0.641)
Bloating	0.140 (0.308)	0.008 (0.967)
Diarrhoea	0.380 (0.004)*	0.050 (0.782)
Constipation	−0.028 (0.839)	0.142 (0.431)
Satiety	0.107 (0.443)	0.210 (0.241)

*Statistically significant

Ulcerative coitis in remission was defined by steroid-free clinical remission, a Mayo Score ≤ 1 on endoscopy, a fecal calprotectin ≤ 200 μg/g and no significant active inflammation on colon biopsies.

*GSRS-IBS* Gastrointestinal symptom rating scale for irritable bowel syndrome

### Correlation between IBS-like symptoms and symptoms of anxiety/depression

There were significant correlations between total GSRS-IBS score and both HADS subscores (anxiety and depression) in the patients with UC in remission (Table 4). The strongest correlation was between anxiety and bloating followed by anxiety-constipation, depression-satiety and anxiety-diarrhoea. In the control subjects there were no significant correlations between GSRS-IBS scores and HADS scores, although there were borderline significant correlations between anxiety-bloating and anxiety-satiety.

**Table 4:** Correlations between irritable bowel syndrome-like symptoms and symptoms of anxiety/depression in patients with ulcerative colitis in remission and in control subjects

	HADS-Anxiety		HADS-Depression	
	Ulcerative colitis in remission (*n* = 55) rs (*p*-value)	Controls (*n* = 33) rs (*p*-value)	Ulcerative colitis in remission (*n* = 55) rs (*p*-value)	Controls (*n* = 33) rs (*p*-value)
Total GSRS-IBS score	0.327 (0.019)*	0.244 (0.194)	0.294 (0.036)*	0.263 (0.160)
Abdominal pain	0.121 (0.394)	0.306 (0.094)	0.148 (0.294)	0.294 (0.109)
Bloating	0.403 (0.003)*	0.347 (0.060)	0.243 (0.079)	0.287 (0.125)
Diarrhoea	0.299 (0.029)*	0.135 (0.470)	0.267 (0.054)	0.207 (0.263)
Constipation	0.380 (0.005)*	-.072 (0.699)	0.258 (0.062)	−0.059 (0.753)
Satiety	0.225 (0.109)	0.333 (0.067)	0.354 (0.010)*	0.182 (0.327)

*Statistically significant

Ulcerative colitis in remission was defined by steroid-free clinical remission, a Mayo Score ≤ 1 on endoscopy, a fecal calprotectin ≤ 200 μg/g and no significant active inflammation on colon biopsies. GSRS-IBS = Gastrointestinal symptom rating scale for irritable bowel syndrome.

*HADS* Hospital anxiety and depression scale

## Discussion

IBS-like symptoms are common in patients with UC with active and inactive disease [5]. However, the present study shows that patients with longstanding disease in remission do not have more IBS symptoms than control subjects. The IBSEN study described the natural course of UC during a five year period after diagnosis, and found that a majority of the patients had a decrease in disease activity over time [3]. Our study may indicate that IBS-like symptoms also diminish over time. Although poorly described in most previous studies, the disease duration in UC patients in remission with IBS-like symptoms has been similar to those without IBS-like symptoms. The reported mean duration of UC disease in previous studies is between 3 and 12 years [7, 8, 14, 26–28], which is shorter than the median disease duration in our UC patients. Another explanation to the low scores on IBS-like symptoms among patients with UC in remission in our study could be the rigorous criteria we used for defining remission. In previous studies there has been a wide heterogeneity for criteria defining remission. A combination of clinical judgement and endoscopic scores to define remission are commonly used [7, 8, 14, 26, 28–33]. In our study, in addition to endoscopy and clinical judgement, we also included a low FC level and the presence of no significant inflammation on the biopsy from the colon to define remission. Perhaps the reported high frequency of IBS in patients with UC in previous studies could partly be explained by the presence of low-grade bowel inflammation. For example, Keohane et al. [7] described that patients with UC in remission and IBS-like symptoms had much higher FC levels than the patients without IBS-like symptoms (591 μg/g vs 229 μg/g). Also Berril et al. [28] found a trend toward a higher median FC in UC patients in clinical remission with IBS-type symptoms. Interestingly, the present study also showed that even in the lower span of FC (≤200 μg/g) there was a moderate significant correlation between FC and symptoms of diarrhoea. One can therefore argue that even in the presence of low-grade inflammation there could be reasons to optimize treatment in patients with UC (especially if they report diarrhoea symptoms).

There could also be alternative explanations to the reported low scores on IBS in patients with longstanding UC. The low degree of symptoms could be due to the adaption at the gut and at the central level leading to “a normalizing of gut discomfort” or down-regulation of gut stimuli as times goes on. In 30–50% of patients with IBS the symptoms decrease or disappear with time [34].

The pathogenesis of IBS is unknown and is probably complex involving multiple mechanisms. Low grade inflammation, intestinal dysbiosis, altered intestinal permeability, visceral hypersensitivity and brain-gut interplay between psychological distress and gut signals have been proposed to be possible mechanisms [35]. In both patients with IBS and in patients with UC in remission there is a relationship between gut symptoms and symptoms of anxiety and depression [27, 31, 32]. In the present study we could nearly confirm an overall correlation of both anxiety and depression scores with IBS scores in the patients with UC in remission.

There are some limitations in our study. Firstly, only 80 out of 216 patients contacted accepted to participate in the study. An unknown number of patients contacted were missed due to reduced personal staff in the summer months at the endoscopy unit. A resistance and inconvenience with leaving a stool sample before the bowel preparation could be a cause for drop-out in some subjects. Secondly, in our study we used the GSRS-IBS questionnaire, which only focuses on symptoms during the last week, and has its major role in screening for IBS-like symptoms in different populations. For a strict IBS diagnosis the Rome III questionnaire is recommended. However, it has been argued that the GSRS-IBS is more sensitive than the Rome III and therefore tends to overestimate the prevalence of IBS [32]. Thirdly, the colonoscopies in our study were performed as clinical routine, and therefore are not rigorously standardized. In addition, the endoscopists may have gotten information from the patients regarding present GI-symptoms and were therefore not sufficiently blinded to the patients’ symptoms.

It is well known that there are sex differences in IBS symptoms with it being more common in women [36]. For example, women report more constipation and somatic discomfort than men. Also, there are sex differences in autonomic and anti-nociceptive responses to visceral stimuli [37]. In our study there were significantly more women in the control group than in the patient group which may have influenced the results towards a higher IBS score in the control subjects. The significantly higher scores of symptoms of constipation in the control group may be contributed to the female dominance in the control group. However, the GSRS-IBS score among the controls in the present study is representative and is similar to that seen in control subjects in other studies [38].

## Conclusions

In conclusion, patients with UC in remission using strict criteria for remission and with longstanding disease do not have more IBS-like symptoms than controls. A moderate correlation exists between FC levels and diarrhoea symptom scores also in UC patients in remission.

## Additional file

Additional file 1: The identities of all subjects are protected in the supporting data set and the file contain data on all parameters included in the manuscript. (XLS 53 kb)

## Abbreviations

FC: Faecal calprotecitn
GSRS-IBS: Gastrointestinal symptoms rating scale-irritable bowel syndrome
HADS: Hospital anxiety depression scale
IBS: Irritable bowel syndrome
UC: Ulcerative colitis
